# Targeting mesothelin receptors with drug-loaded bacterial nanocells suppresses human mesothelioma tumour growth in mouse xenograft models

**DOI:** 10.1371/journal.pone.0186137

**Published:** 2017-10-23

**Authors:** Mohamed A. Alfaleh, Christopher B. Howard, Ilya Sedliarou, Martina L. Jones, Reema Gudhka, Natasha Vanegas, Jocelyn Weiss, Julia H. Suurbach, Christopher J. de Bakker, Michael R. Milne, Bree A. Rumballe, Jennifer A. MacDiarmid, Himanshu Brahmbhatt, Stephen M. Mahler

**Affiliations:** 1 Australian Institute for Bioengineering and Nanotechnology (AIBN), The University of Queensland, Brisbane, Queensland, Australia; 2 Faculty of Pharmacy; King Abdulaziz University, Jeddah, Saudi Arabia; 3 Centre for Advanced Imaging, The University of Queensland, Brisbane, Queensland, Australia; 4 Australian Research Council Training Centre for Biopharmaceutical Innovation, The University of Queensland, Brisbane, Queensland, Australia; 5 Cancer Therapeutics, EnGeneIC Ltd, Sydney, New South Wales, Australia; 6 Queensland Brain Institute (QBI), The University of Queensland, Brisbane, Queensland, Australia; Wayne State University, UNITED STATES

## Abstract

Human malignant mesothelioma is a chemoresistant tumour that develops from mesothelial cells, commonly associated with asbestos exposure. Malignant mesothelioma incidence rates in European countries are still rising and Australia has one of the highest burdens of malignant mesothelioma on a population basis in the world. Therapy using systemic delivery of free cytotoxic agents is associated with many undesirable side effects due to non-selectivity, and is thus dose-limited which limits its therapeutic potential. Therefore, increasing the selectivity of anti-cancer agents has the potential to dramatically enhance drug efficacy and reduce toxicity. EnGeneIC Dream Vectors (EDV) are antibody-targeted nanocells which can be loaded with cytotoxic drugs and delivered to specific cancer cells via bispecific antibodies (BsAbs) which target the EDV and a cancer cell-specific receptor, simultaneously. BsAbs were designed to target doxorubicin-loaded EDVs to cancer cells via cell surface mesothelin (MSLN). Flow cytometry was used to investigate cell binding and induction of apoptosis, and confocal microscopy to visualize internalization. Mouse xenograft models were used to assess anti-tumour effects *in vivo*, followed by immunohistochemistry for *ex vivo* evaluation of proliferation and necrosis. BsAb-targeted, doxorubicin-loaded EDVs were able to bind to and internalize within mesothelioma cells *in vitro* via MSLN receptors and induce apoptosis. In mice xenografts, the BsAb-targeted, doxorubicin-loaded EDVs suppressed the tumour growth and also decreased cell proliferation. Thus, the use of MSLN-specific antibodies to deliver encapsulated doxorubicin can provide a novel and alternative modality for treatment of mesothelioma.

## Introduction

Human malignant mesothelioma is an aggressive tumour that develops from mesothelial cells, which line the pleura, peritoneum and the pericardium [1]. Patients with malignant mesothelioma suffer from severe symptoms, which significantly affect the quality of their life [2]. Malignant mesothelioma is commonly attributed to exposure to asbestos, which consists of a group of fibrous needle-like silicates [3]. Asbestos exposure causes random chromosomal damage, leading to an aberrant activation of different autocrine pathways in the genotoxic damaged cells, which increases cell survival and promotes neoplastic cell migration into other host tissues [4, 5]. Asbestos fibres lead to generation of free radicals that connect the inflammatory responses and the initiation and progression of mesothelioma by damaging mesothelial cell DNA [6, 7].

According to the World Health Organization (WHO), 125 million workers are exposed to asbestos on a daily basis and it is estimated that 107,000 deaths occur annually due to asbestos-related diseases [8]. Notwithstanding the known risks of exposure, asbestos is still used in many developing countries around the world. The median latency between asbestos exposure and disease onset is around 44.6 years, which makes mesothelioma difficult to diagnose and treat in its early stages [9]. The incident rates are escalating, with peak incidences expected from 2020 [10].

Intensive treatment plans for early stage patients, which include surgery, chemotherapy and radiotherapy, must be conducted to treat the tumour and avoid its recurrence. However, not all patients are eligible for surgery and therapeutic doses of radiation are associated with serious toxicity [11, 12]. Although the available chemotherapeutic agents have limited value in malignant mesothelioma treatment, the FDA approved cisplatin-pemetrexed as a first-line therapy in unresectable patients [13], and has resulted in an improvement in the response rates and overall survival rates in clinical trials [14, 15]. Nevertheless, the median overall survival rate with this combination therapy is only one year [16], and it is therefore vital to develop more effective anti-mesothelioma agents.

Chemotherapy, although being effective in slowing tumour growth and in some cases stabilizing metastases, has major drawbacks, including: limited drug potency, non-selectivity and development of multi-drug resistance (MDR). EnGeneIC Dream Vectors (EDVs) are targeted and drug loaded 400 ± 20 nm, anucleate nanocells derived from gram-negative *Salmonella typhimurium*, produced as a consequence of mutation of the genes that control bacterial cell division [17–20]. Chemotherapeutic agents with different properties can be effectively packaged within the EDV nanocells at significant concentrations (up to 10 million drug molecules per nanocell) [20].

Targeted delivery was achieved by using tumour-specific monoclonal antibodies (mAbs) reformatted as bispecific antibodies (BsAbs), which are capable of binding the EDVs with one arm and the tumour antigen with the other arm [21]. Safety of the EDV delivery system has been investigated in different animal models: mice [20], monkeys [22] and dogs [23]. The EDVs proved to be safe and well tolerated despite high and repeated doses. Recently, open label, multicentre phase I human clinical trials were performed to evaluate the safety of EDV nanocells targeting epidermal growth factor receptor (EGFR) [24, 25]. Overall, EDV nanocells were deemed safe, and the most common treatment-related adverse events were nausea, transient and self-limiting rigors and fever. Additionally, EDVs showed an ability to overcome multiple-drug resistance (MDR) in tumours through a two-wave treatment approach: firstly, to deliver EDV packaged with siRNA or plasmid-encoded shRNA to suppress the specific MDR gene *in vivo*; secondly, to deliver EDVs loaded with a cytotoxic agent(s) [26, 27].

Malignant mesotheliomas frequently express mesothelin (MSLN) [28, 29], an immunogenic glycosylphosphatidylinositol (GPI)-anchored cell surface protein. Studies show that MSLN plays a role in cell adherence [30], survival, proliferation, tumour progression [31–34] and chemotherapy resistance [34–37]. An *in vitro* evaluation revealed that MSLN increased cancerous cell proliferation significantly, and silencing the MSLN gene decreased cancer cell proliferation, migration and invasiveness [31, 32]. Accordingly, MSLN has been identified as a target for immunotherapy for human malignant mesothelioma.

Amatuximab and HN1 are both anti-MSLN mAbs with potential to be utilised for treatment of human malignant mesothelioma [38–41]. Amatuximab was developed as a high affinity chimeric mAb (K_D_ 1.5 nM), that comprises murine anti-MSLN SS1 scFv (K_D_ 11 nM) [39, 42] and human constant regions [38]. The preclinical evaluation showed the efficacy of amatuximab in combination with gemcitabine or paclitaxel (Taxol) in a treatment of MSLN-positive xenografts [38]. A phase II clinical trial of amatuximab in combination with pemetrexed and cisplatin resulted in a disease control rate of 90% and improved the median overall survival in comparison to a phase III clinical trial of cisplatin and pemetrexed alone [15, 43]. The HN1 scFv was isolated from a naïve, human scFv phage library and had an affinity of K_D_ 100 nM [40]. The scFv was engineered as a human IgGγ1κ mAb, which improved the affinity 30-fold (K_D_ 3 nM) [40].

**Fig 1:**
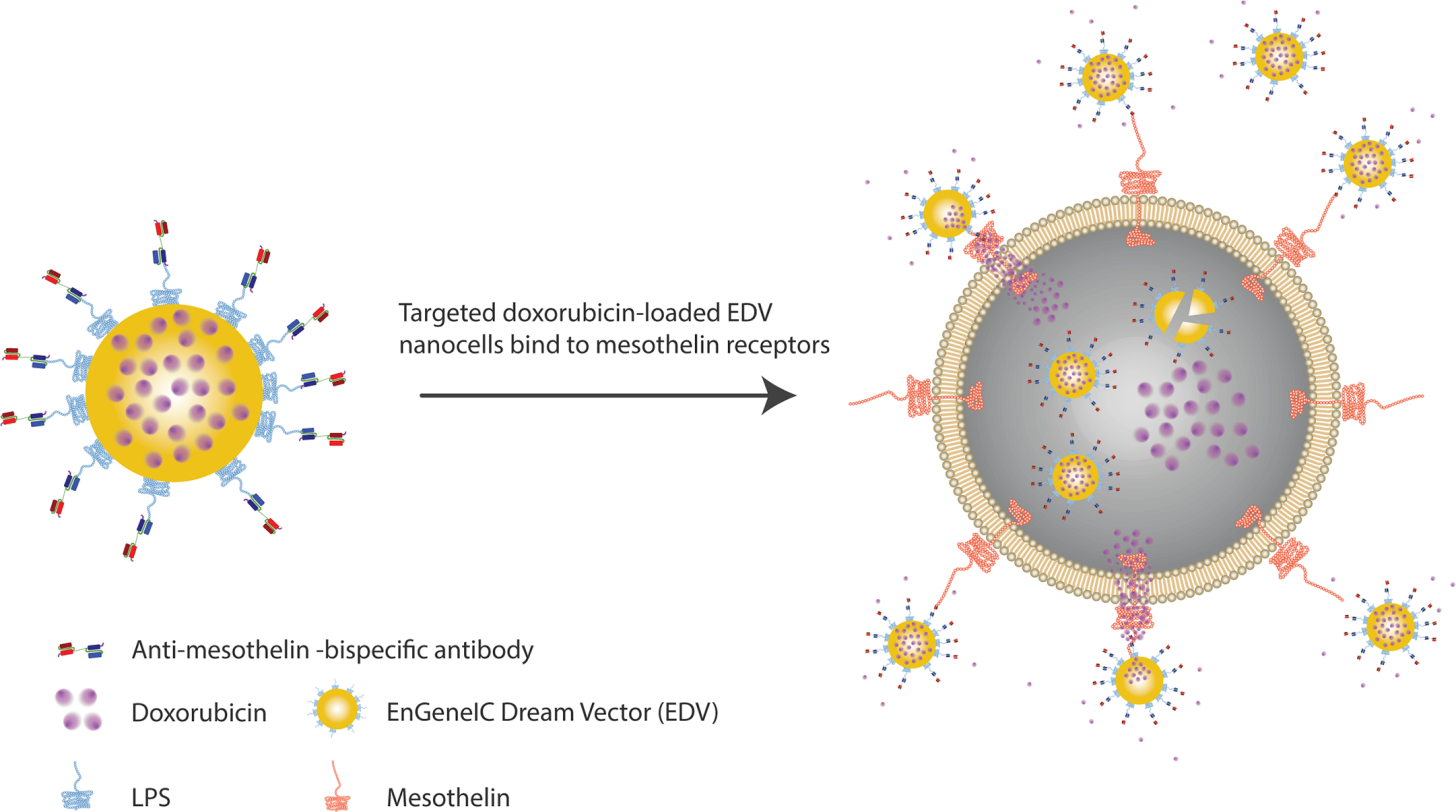
Schematic representation showing the mechanism of ^Anti-MSLN-BsAb^EDV_Dox_ nanocell mediated intracellular doxorubicin delivery. Active binding of the EDV nanocells (yellow) to the MSLN cell-surface receptor (red) occurs via the BsAb. The EDV is lysed intracellularly to release the doxorubicin (purple) into the cell cytoplasm.

In this study, we evaluated whether anti-MSLN BsAbs could be used to specifically deliver drug-loaded EDVs to mesothelioma and suppress tumour growth in mesothelioma xenografts (Fig 1). Therefore, we reformatted amatuximab and HN1 scFvs as BsAbs to target EDV to MSLN-positive tumours. The BsAb format utilized a glycine-serine (G_4_S) linker to connect two scFvs, whereby one scFv binds to the lipopolysaccharide antigen (LPS) on the EDV surface and the other binds to the target receptor, as reported by Taylor et al. [21]. The amatuximab-derived BsAb (Amatux-BsAb) in conjugation with doxorubicin (Dox)-loaded EDV nanocells (^Amatux^EDV_Dox_) was capable of binding human mesothelioma cells *in vitro*, resulting in a release of doxorubicin intracellularly and induction of apoptosis. Additionally, in human malignant mesothelioma xenograft mouse models, treatment with ^Amatux^EDV_Dox_ led to statistically significant suppression of tumour growth, measured by both tumour size and a reduction in nuclear staining of excised tumour by Ki67, an antibody which recognizes a marker of cell cycle proliferation [44–46]. These results suggest that the Amatux-BsAb in conjugation with doxorubicin loaded EDV nanocells are effective despite using significantly lower doses of the drug, offering a potential new avenue for malignant mesothelioma-targeted therapy.

## Methods

### Protein expression of anti-MSLN-LPS BsAbs

**Fig 2:**
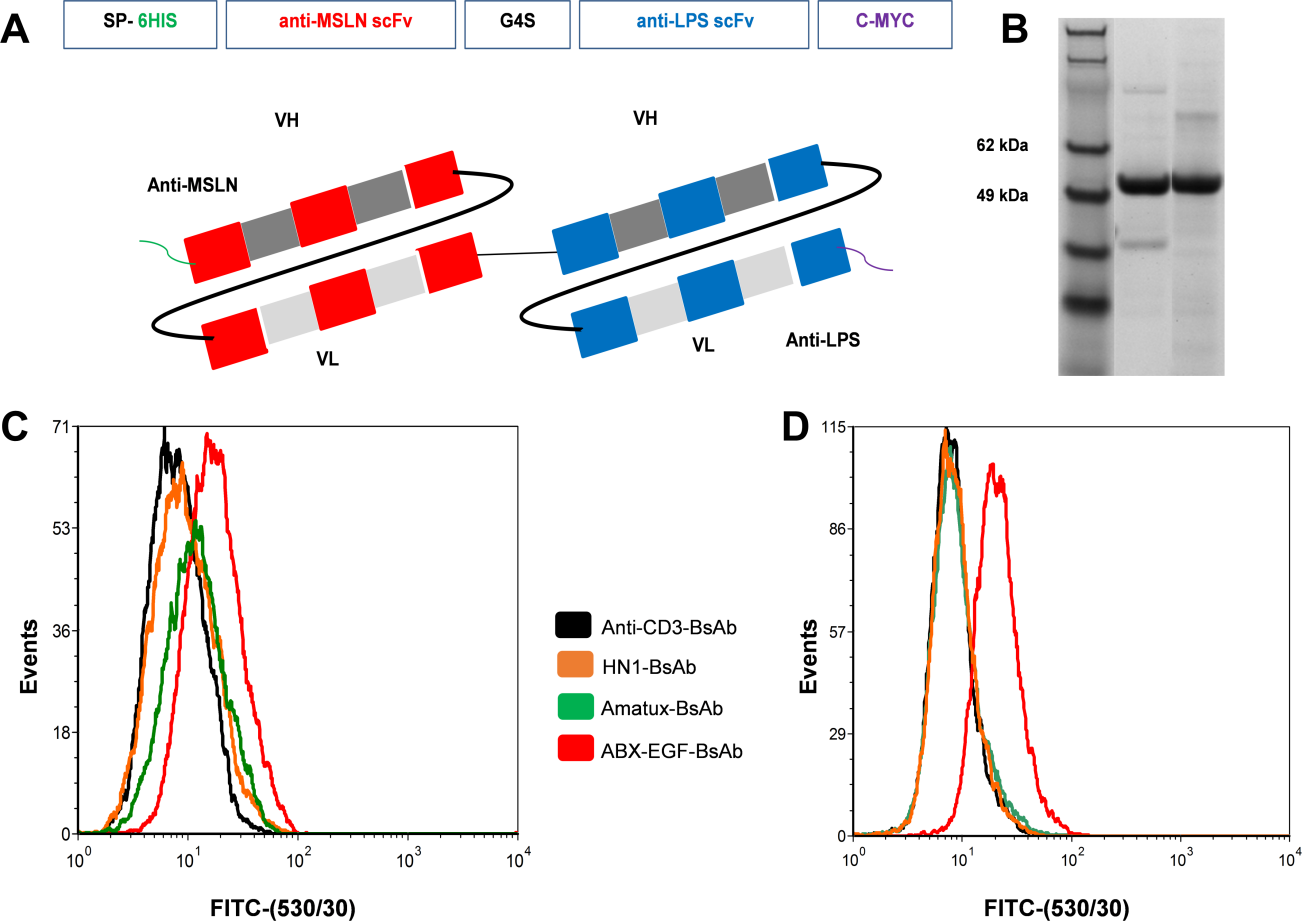
Anti-MSLN-LPS BsAb gene design, synthesis and binding to human mesothelioma cell lines. (A) A BsAb comprising anti-MSLN scFv (red) and anti-LPS scFv (blue), connected by a glycine-serine linker (G4S). 6-HIS and C-MYC was included into the design for purification and detection purposes. Secretion peptide (SP) was included to enable the BsAbs to be secreted from CHO cells into medium. (B) SDS- PAGE gel showing, starting from left, SeeBlue pre-stained standard MW marker (Invitrogen) (Lane 1), Amatux-BsAb (Lane 2) and HN1-BsAb (Lane 3) purified products (Both MWs are 56 kDa). Flow cytometry was used to test the anti-MSLN designed BsAbs and an anti-EGFR control BsAb (ABX-EGF-BsAb) binding to human mesothelioma cell lines, (C) H226 and (D) MSTO-211H. Binding of anti-CD3-BsAb (black line), ABX-EGF-BsAb (red line), Amatux-BsAb (green line) and HN1-BsAb (orange line) to cells was detected using FITC-conjugated anti-c-myc antibody.

Both anti-MSLN amatuximab [IMGT/mAb-DB ID 64] and HN1 [40] were reformatted as BsAbs each with an anti-LPS scFv to allow binding to MSLN and to LPS. The sequences were codon-optimized for expression in Chinese hamster ovary (CHO) cells. The sequence of each BsAb included a mammalian leader sequence from immunoglobulin kappa chain, followed by a 6x Histidine purification tag, then the anti-MSLN scFv sequence, a G_4_S linker, the anti-LPS scFv sequence and a C-terminal myc-tag for detection (Fig 2A) as described in Taylor et al. [21]. Both Amatux-LPS and HN1-LPS BsAbs were cloned into the mammalian expression vector pcDNA3.1(+) (Life Technologies), using *Hind*III and *Not*I restriction sites. CHO-S cells (Life Technologies) were cultured in CD-CHO with 8 mM GlutaMAX (Gibco) at 37°C, 7.5% CO_2_ with shaking at 130 rpm. On the day of transfection, cells were prepared at ~ 3.0 × 10^6^ cells/mL at 98% viability as measured using Cedex HiRes cell counter. The transient transfection for 200 mL culture was prepared as following: 600 μg of plasmid DNA was mixed with 15 mL OptiPRO serum free media (SFM) (Gibco) (15% total volume) and incubated at room temperature (RT) for 30–60 sec. Concurrently, 2400 μL of PEIpro (Polyplus-transfection) was mixed with 30 mL OptiPRO SFM (15% total volume) and incubated at RT for 30–60 sec. The two solutions were then mixed together by gently pipetting the PEIpro: OptiPRO complex into the plasmid DNA: OptiPRO complex, and incubated static at RT for 15 mins. The transfection complex was then added to the culture in a shake-flask and mixed by gentle swirling. The culture was incubated at 37°C, 7.5% CO_2_ with shaking at 130 rpm for 4 to 6 hrs, after which the culture was diluted 1:2 (v:v) with CD-CHO supplemented with final concentrations of EfficientFeed A and B (Gibco) 7.5% (v/v), 0.4% Anti-Clumping Agent (Gibco), 8 mM GlutaMAX and then the flasks were kept shaking at 130 rpm at 32°C, 7.5% CO_2_ for 12 days. The supernatant was then harvested and 0.22 μm filtered.

### Protein purification

Both His-tagged BsAbs were purified using immobilized metal affinity chromatography with a 5 mL HisTrap excel column (GE Healthcare). The column was equilibrated in 20 mM sodium phosphate pH 7.4, 500 mM NaCl. The harvested supernatant was loaded onto the column and then washed with equilibration buffer containing 20 mM imidazole to remove non-specifically bound contaminants. The protein of interest was then eluted with 20 mM sodium phosphate pH 7.4, 500 mM NaCl, 500 mM imidazole. The final product was desalted into PBS using HiPrep 26/10 column (GE Healthcare) and then filtered through a 0.22 μm syringe filter. The concentration of protein was determined by measuring the UV absorbance at 280 nm using a Nanodrop 1000 spectrophotometer. Protein purity was analysed by SDS-PAGE using 4–12% Bis-Tris gels (Invitrogen).

### Binding analysis of BsAbs to human mesothelioma cells by flow cytometry

To evaluate the binding affinity of anti-MSLN BsAbs to MSLN receptors on the human lung mesothelioma cell lines H226 (ATCC, CRL-5826) and MSTO-211H (ATCC, CRL-2081), cells were grown in RPMI-1640 (Sigma) medium supplemented with 10% fetal calf serum (FCS; Bovogen) and Penicillin/Streptomycin (Sigma). Before flow cytometric analysis, the cells were washed twice with PBS and then scraped from T75 flask and centrifuged at 700 x g for 5 mins. The viable cells were counted by trypan blue exclusion method and 8x10^6^ cells/mL were washed twice with cold PBS. A centrifugation step at 700 x g was conducted in between the washes. The sample was divided into five vials (2x10^6^ cells/sample) prior to the final centrifugation step. The pellets were resuspended in 500 μμL Binding Buffer (PBS containing 0.1% sodium azide and 5% bovine serum albumin (BSA)) and incubated for 10 mins to block nonspecific binding sites. Various primary antibodies (4 μg) were added to each tube as follows: (1) Amatux-BsAb, (2) HN1-BsAb, (3) ABX-EGF-BsAb (anti-EGFR BsAb described in [21]), (4) Anti-CD3-BsAb (as a negative control). The tubes were incubated for 45 mins on ice on a gentle shaker. Then the cells were washed twice with Binding Buffer. The cells were incubated with the secondary antibody, anti-cMyc-FITC (Miltenyi Biotec), diluted 1 in 250 in Binding Buffer for 45 mins on ice on a shaker (moderate movement). Afterwards, the cells were washed three times with Binding Buffer, and re-suspended in 400 μL PBS for flow cytometric analysis on a Beckman Coulter, Cytomics CXP500 flow cytometer using the 488 nm laser and the 530/30 nm filter. The data were analysed using FCS Express Flow version 4 (De Novo Software).

### Preparation of targeted nanocells

EDVs were manufactured and purified using a cross-flow filtration and lyophilized as previously described [20]. Each lyophilized vial contained 1.1x10^10^ EDVs and the lyophilized particles were resuspended in 600 μL of solvent resulting into 1.8x10^10^ EDVs/mL. Doxorubicin loading into EDV nanocells to create EDV_Dox_ was carried out as previously described [20]. Additionally, for some experiments EDV nanocells were labelled with Alexa-Fluor 488 (AF-488) (ThermoFisher) as previously described [21]. Drug-loaded or AF-488 labelled EDVs were then incubated with Amatux-BsAb at RT for 30 mins while shaking at 300 x g, to create ^Amatux^EDV. Samples then underwent 3x PBS spin wash cycles at 9,000 x g for 8 mins each to remove any excess Amatux-BsAb. ^Amatux^EDV nanocells were resuspended to concentrations as required for the experiment.

### Internalization analysis of drug-loaded ^BsAb^EDV nanocells into H226 cells by confocal microscopy

H226 cells were incubated on coverslips with AF-488 labelled ^Amtaux^EDV_Dox_, at a ratio of 10,000 EDV nanocells per cell. Cells without any treatment were used as a control. Cells were incubated at 37°C for 3 hrs and 24 hrs which was followed by 3 washes in sterile PBS. Cells were fixed with 4% paraformaldehyde for 10 mins, followed by 3 times PBS washes. After the final wash, 500 μL PBS was added to each well to cover the coverslips completely. An anti-EGFR mAb (528 mAb) was labelled with x-site fluor 670/755 (Kodak) and used as a cell membrane stain; 4 μg was added directly to each sample. Wells were mixed gently and left for 10 mins to allow cells to stain sufficiently. All coverslips were again washed 3 times with PBS followed by a final wash in Milli-Q water and then mounted on clean slides using Fluka Eukitt (Sigma) quick-hardening mounting medium. Cells were imaged using a Carl Zeiss 710 Inverted Laser Scanning Confocal Microscope housed at the Australian National Fabrication Facility, Queensland Node (ANFF) using a plan-apochromat 63x 1.40 Oil DIC M27 objective and ZEN 2008 software for image formatting. The AF-488 labelled EDV nanocells were detected using ex488/em525 nm. Doxorubicin is auto-fluorescent and detected using ex490/em560 nm. Cell surface EGFR was detected with ex633/em670 nm.

### Binding analysis of ^Amatux^EDV_Dox_ on human mesothelioma cell by flow cytometry

To evaluate binding of ^Amatux^EDV_Dox_ to MSLN receptors on H226 and MSTO-211H cells, lyophilised EDV_Dox_ nanocells were reconstituted, loaded with doxorubicin, and then labelled with Amatux-BsAb or with anti-Murine Epithelial Cell Adhesion Molecule (mEpCAM)-BsAb as a negative control. The ^BsAb^EDv_Dox_ complexes were incubated rotating for 1 hr at RT, before washing three times and resuspending in 500 μL of PBS. H226 and MSTO cells were grown as previously described. Cells were washed twice with PBS and then detached using Accutase (Sigma) for 10 mins at 37°C and centrifuged at 300 x g for 5 mins. Viable cells were counted by trypan blue exclusion method on a hemocytometer and 1x10^7^ cells/mL were resuspended in serum free media. The cell suspension was divided into three vials to be treated with: (1) ^Non-targeted^EDV_Dox_, (2) ^mEpCAM^EDV_Dox_, and (3) ^Amtaux^EDV_Dox_. EDVs (5x10^9^ EDV/sample) were added to 5x10^5^ cells (1:10,000 cell:EDV ratio) and incubated rotating at 4°C. After 2 hrs, unbound EDVs were removed by washing the cells with PBS+1%BSA at 300 x g for 5 mins. Cells were fixed with 4% paraformaldehyde for 10 mins at RT, followed by a PBS+1%BSA wash. Bound EDVs were stained with 1 μg of anti-LPS mAb (Biodesign International) labelled with AF-488 (Life Technologies) for 45 mins at RT in the dark. The cells were washed three times with PBS+1%BSA at 300 x g for 5 mins, before analysis on a Beckman Coulter, Gallios flow cytometer using the 488 nm laser and the 530/30 nm filter. The data were analysed using FCS Express Flow version 4 (De Novo Software).

A flow cytometry dose-response of ^Amatux^EDV binding to the aforementioned mesothelioma cell lines, and the murine colon carcinoma CT26 cells (ATCC, CRL-2638), was performed in similar manner, but without loading the EDVs with doxorubicin. The cell suspensions were treated with different amounts of ^Amatux^EDV (1:1000, 1:2500, 1:5000 and 1:10,000 cell: ^Amatux^EDV ratios). EDVs charged with anti-CD3 BsAb were also included as negative controls when we incubated the cell suspensions with constant concentration of ^CD3^EDV (1:10,000 cell:^CD3^EDV). The data were analysed using FCS Express Flow version 4 (De Novo Software).

### Cytotoxicity of ^Amatux^EDV_Dox_ nanocells

Induction of apoptosis was demonstrated by staining cells with Annexin V-FITC after incubating MSLN-positive H226 cells with ^Amatux^EDV, ^Non-targeted^EDV_Dox_ and ^Amatux^EDV_Dox_. H226 cells were grown in DMEM (Sigma) with 10% FCS (Bovogen) and 1% Penicillin/Streptomycin at a seeding density of 3x10^5^ cells per well in 6 well plates. The cells were incubated with ^Amatux^EDV, ^Non-targeted^EDV_Dox_ and ^Amatux^EDV_Dox_ at a ratio equivalent to 10,000 EDVs per cell, for 2 h at 37°C to allow EDVs to bind to cells. Then the cells were washed 3 times with PBS to remove unbound EDVs, before adding fresh media and incubating at 37°C for 72 hrs to allow EDV internalisation and doxorubicin release. Washing the unbound particles is necessary, because if left in a well more than 24 hrs, they will start to leak out the doxorubicin into media [20, 22]. After 72 hrs, the media were collected and the cells were detached with trypsin-EDTA and collected in media. The cells were washed twice in PBS, then resuspend in 1x Binding Buffer at 1x10^6^ cells in 1 mL. Each sample was divided equally (500 μL) into different tubes, with one treated with 5 μL of Annexin V-FITC while the other was treated with 5 μL of Binding buffer as a control for doxorubicin interference by autofluorescence. The tubes were incubated at RT for 10 min before analysing the fluorescence of the cells on a Beckman Coulter, Cytomics CXP500 flow cytometer, using the 488 nm laser and the 530/30 nm filter. Data were analysed using FCS Express 4 Flow cytometry (De Novo Software).

### Effect of ^Amatux^EDV_Dox_ nanocells on tumour regression

The experiments associated with the effect of Amatux-targeted nanocells on tumour regression were performed essentially as described by Taylor et al. [21]. H226 mesothelioma cells were grown in RPMI with 10% FCS and penicillin/streptomycin. Six week-old female Balb/c mice were injected subcutaneously on the left flank with 5×10^6^ cells in 200 μL of media together with 200 μL of growth factor reduced matrigel (BD Biosciences). Once the tumours reached ~110 mm^3^ (Day 11) the mice were randomized into treatment groups which included 5–6 mice per group, as follows: Group 1 –Saline, Group 2 −^Amatux^EDV, Group 3 −^Non-targeted^EDV_Dox_, and Group 4 −^Amatux^EDV_Dox_. Each mouse was intravenously injected with 1×10^9^ of the relevant EDVs administered 3 times per week for 3 weeks, and tumour size was monitored throughout the treatment period up to Day 39 post xenograft. The mice were weighed twice a week for 3 weeks, as per previous methodology [20, 27]. A two-sample t-test with equal variance was performed to directly compare the mean tumour volume from each individual treatment to determine statistical significance between treatments and control. Statistical significance was determined as p ≤ 0.05 [47]. The mice were euthanased after a total of 12 injections, tumours were harvested, paraformaldehyde fixed and embedded in paraffin. Note; Experiments were conducted in compliance with the Australian National Health and Medical Research Council guidelines for the care and use of laboratory animals and with the approval of the EnGeneIC Animal Ethics Committee.

### Ki67 cell staining and quantification by immunohistochemistry

Paraffin embedded human mesothelioma tumour tissue section (5 μm), prepared from tumours excised from the four xenograft treatment groups at Day 39 (Saline, ^Amatux^EDV, ^Non-targeted^EDV_Dox_, ^Amatux^EDV_Dox_) were dewaxed in xylene, rehydrated in graded alcohols and deionized in distilled water. Heat-induced antigen retrieval (HIER) was performed by immersing the slides for 20 mins in 0.01 M Citric Acid-Based Buffer, pH 6.0 (Vector), at 100°C in a microwave, followed by cooling for 30 mins at RT. The endogenous peroxidase activity was quenched by incubating the slides with methanol containing 1% hydrogen peroxide for 30 min at RT in the dark. Then the slides were washed using PBS with 1% Tween20 (PBS-T) for 5 min and then blocked with 5% goat serum in PBS-T for 30 min at RT. Rabbit anti-human pKi67 antibody (Sino Biological, US) was added to the slides at 20 μg/μL. PBS substituted the primary antibody for the control slide. The slides were incubated overnight at 4°C in a covered humidity chamber. On the next day, the slides were washed in PBS-T and the HRP-conjugated secondary antibody goat anti-rabbit IgG (Life Technologies) was diluted in 5% goat serum in PBS-T at 1:200 before it was incubated with the slides for 2 hrs at RT. Then the slides were washed 3 times in PBS-T, then incubated with AEC (3-amino-9-ethylcarbazole) substrate solution (Vector) until the desired colour developed (10 min). The slides were counterstained with hematoxylin (Blue) for 30 sec and mounted with VectaMount AQ (Vector). The percentage of Ki67 positive cells was estimated by blind counting of Ki67 positive cells (red nuclei) in two different Ki67 expressing regions consisting of 1000 cells in total using 20x magnification. The percentage was estimated from three different mice representing each group. Images were captured using Aperio digital pathology slide scanner and analysed using Aperio ImageScope software (Leica Biosystems). The mean values for the percentage of Ki67 positive cells for all treatments were analysed for variance using one-way ANOVA with Tukey post-hoc test was performed to directly compare the means from each individual treatment to determine if there is any significant difference between treatments and control. Statistical significance was determined as p ≤ 0.05 [47].

### Hematoxylin and Eosin (H&E) staining for mesothelioma paraffin embedded tumour samples

Paraffin embedded human mesothelioma tumour tissue sections (5 μm), prepared from tumours excised from the four xenograft treatment groups at Day 39 (Saline, ^Amatux^EDV, ^Non-targeted^EDV_Dox_, ^Amatux^EDV_Dox_) were dewaxed in xylene, rehydrated in graded ethanol solutions. Tissue samples were then stained for 7 min in Mayers Haematoxylin and 20 sec in 1% Eosin. Samples were then dehydrated through graded ethanol solutions and cleared with xylene. Finally, slides were coverslipped using DPX solution (Sigma). Samples were visualised using an upright bright-field slide scanner (Zeiss Axio Imager Z2). The percentage of necrotic areas was estimated by blind measuring of multiple scattered necrotic regions (absence of nuclear staining) using 20x magnification. The percentage was estimated from three different mice representing each treatment group. Images were captured using a Zeiss Axio Imager Z2 slide scanner and analysed using ImageJ software v1.50i (National Institutes of Health, Bethesda, MD). The mean values for the percentage of necrotic regions for all treatments were analysed for variance using one-way ANOVA with Tukey post-hoc test. Statistical significance was determined as p ≤ 0.05 [47].

## Results

### Production of anti-MSLN BsAbs

The BsAbs derived from amatuximab and HN1 were expressed in CHO cells followed by IMAC purification using HisTrap excel affinity chromatography column. SDS-PAGE analysis showed molecular weights of 56 kDa for both BsAbs (Fig 2B). Binding specificity between purified MSLN and purified LPS and the BsAbs was confirmed by conducting indirect ELISA on immobilised MSLN and LPS and detection with HRP-conjugated anti-myc antibody (data not shown).

### Binding activity of anti-MSLN BsAbs toward human mesothelioma cells

Flow cytometry was used to assess the binding of Amatux-BsAb and HN1-BsAb to mesothelioma cell lines H226 and MSTO-211H. ABX-EGF-BsAb specific for EGFR [21] was used as a positive control, since malignant mesothelioma frequently shows over-expression of EGFR in either cell lines or the tumour itself [29]. We observed that Amatux-BsAb and ABX-EGFR-BsAb bound strongly to the H226 cells, whereas HN1-BsAb showed no binding (Fig 2C). Both Amatux and HN1-BsAbs showed no binding to MSTO-211H cells (Fig 2D).

### ^Amatux^EDV_Dox_ nanocells cellular uptake

**Fig 3:**
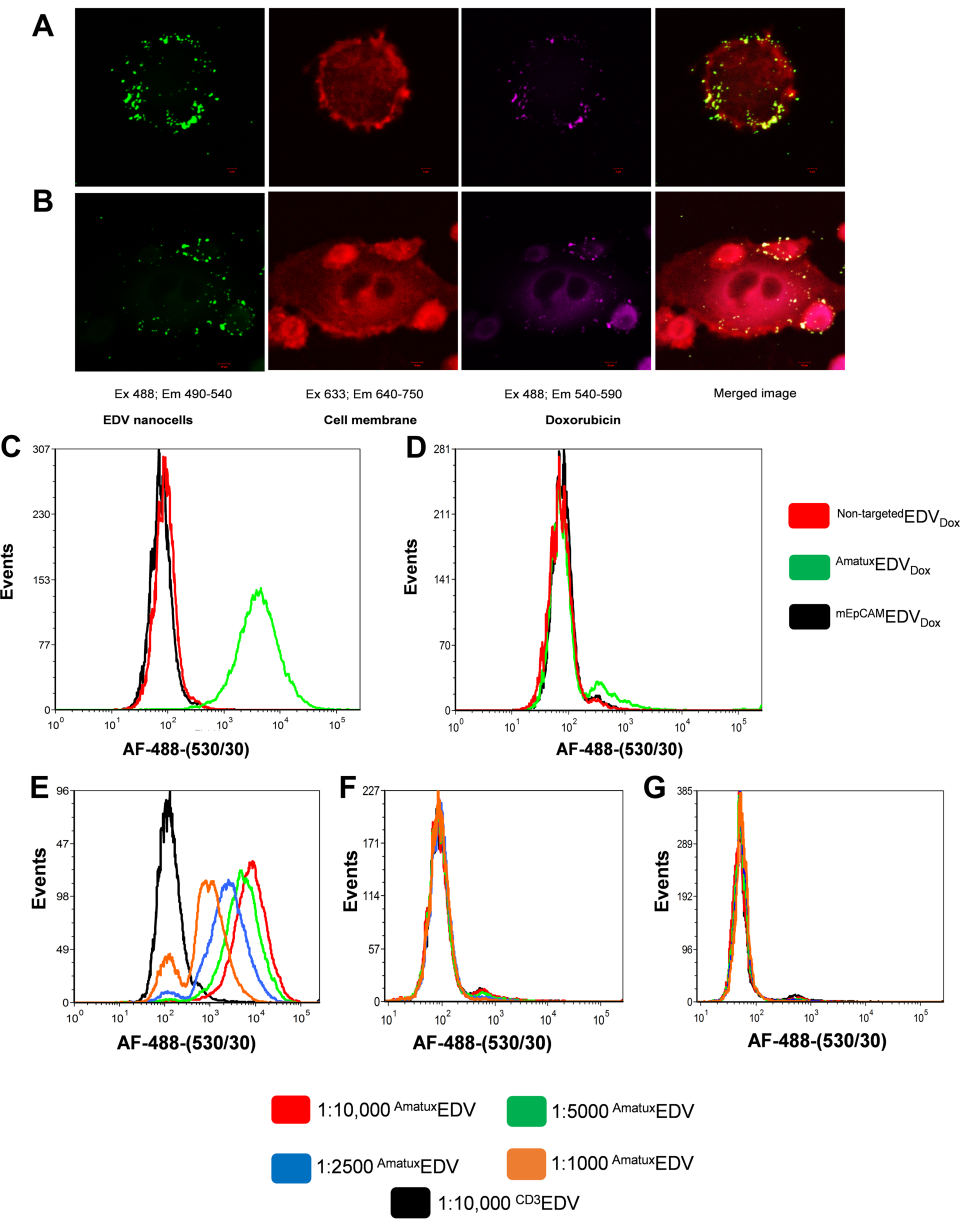
BsAb targeting and internalisation of EDV_Dox_ to MSLN on H226 cells. Internalisation was detected using confocal imaging of H226 cells incubated with Amatux-BsAb targeted AF-488-EDV nanocells (Green: Ex 488; Em 490–540) loaded with doxorubicin (Purple: Ex 488; Em 540–590) at 3 hr (A) and 24 hr (B) timepoints (Scale bars 5 and 10 μm, respectively). H226 cell membranes were pre-stained with anti-EGFR-AF-647 (Red: Ex 633; Em 640–750). Specificity of ^Non-targeted^EDV_Dox_ (red line), ^mEpCAM^EDV_Dox_ (black line) and ^Amatux^EDV_Dox_ (green line) binding to human mesothelioma cell lines, H226 (C) and MSTO-211H (D), and dose-response assay of ^Amatux^EDV_Dox_ on H226 (E), MSTO-211H (F) and CT26 (G) were excuted by flow cytometry using anti-LPS AF-488 and 488 nm laser and 530/30 nm filter.

^BsAb^EDV_Drug_ nanocells are capable of being internalized by cancer cells and releasing cytotoxic drug intracellularly [22]. Here, we prepared AF-488 labelled ^Amatux^EDV_Dox_ nanocells to monitor and visualize binding and internalisation of the targeted EDV system to H226 cells at the cellular level by confocal microscopy. Within a 3 hr time-frame, the ^Amatux^EDV_Dox_ nanocells were co-localized on the cell surface, demonstrated by green fluorescence staining on the H226 cell membrane (Fig 3A). After 24 hrs of incubation, the AF-488 labelled ^Amatux^EDV_Dox_ nanocells migrated and accumulated within the H226 cells and released doxorubicin intracellularly, demonstrated by purple staining representing doxorubicin autofluorescence (Fig 3B). To confirm that the ^Amatux^EDV nanocells binding to H226 cells was Amatux-BsAb-dependent, we used flow cytometry to test binding of ^Non-targeted^EDV_Dox_, ^mEpCAM^EDV_Dox_ and ^Amatux^EDV_Dox_ to H226 and MSTO-211H cell lines_._ Only ^Amatux^EDV_Dox_ nanocells were able to bind to the MSLN on H226 cells, but did not bind to the low MSLN expressing cell line MSTO-211H (Fig 3C and 3D).

A dose-response shift was generated when H226 mesothelioma cells were treated with different concentrations of ^Amatux^EDV (Fig 3E). The intensity of the binding shift was concentration-dependent, wherein the strongest shift was observed when the H226 cells were treated with 10,000 ^Amatux^EDV per cell, while a gradual reduction of the binding intensity was observed each time we reduced the ^Amatux^EDV:cell ratio by half (Fig 3E). Under the same conditions, ^Amatux^EDV showed no binding to both MSTO-211H and murine colon carcinoma CT26 cells (MSLN negative cell line) (Fig 3F and 3G). Also, as a negative control we included 1:10,000 cell:^CD3^EDV, which showed no binding to the three cancer cell lines.

### Cytotoxicity of ^Amatux^EDV_Dox_ nanocells

**Fig 4:**
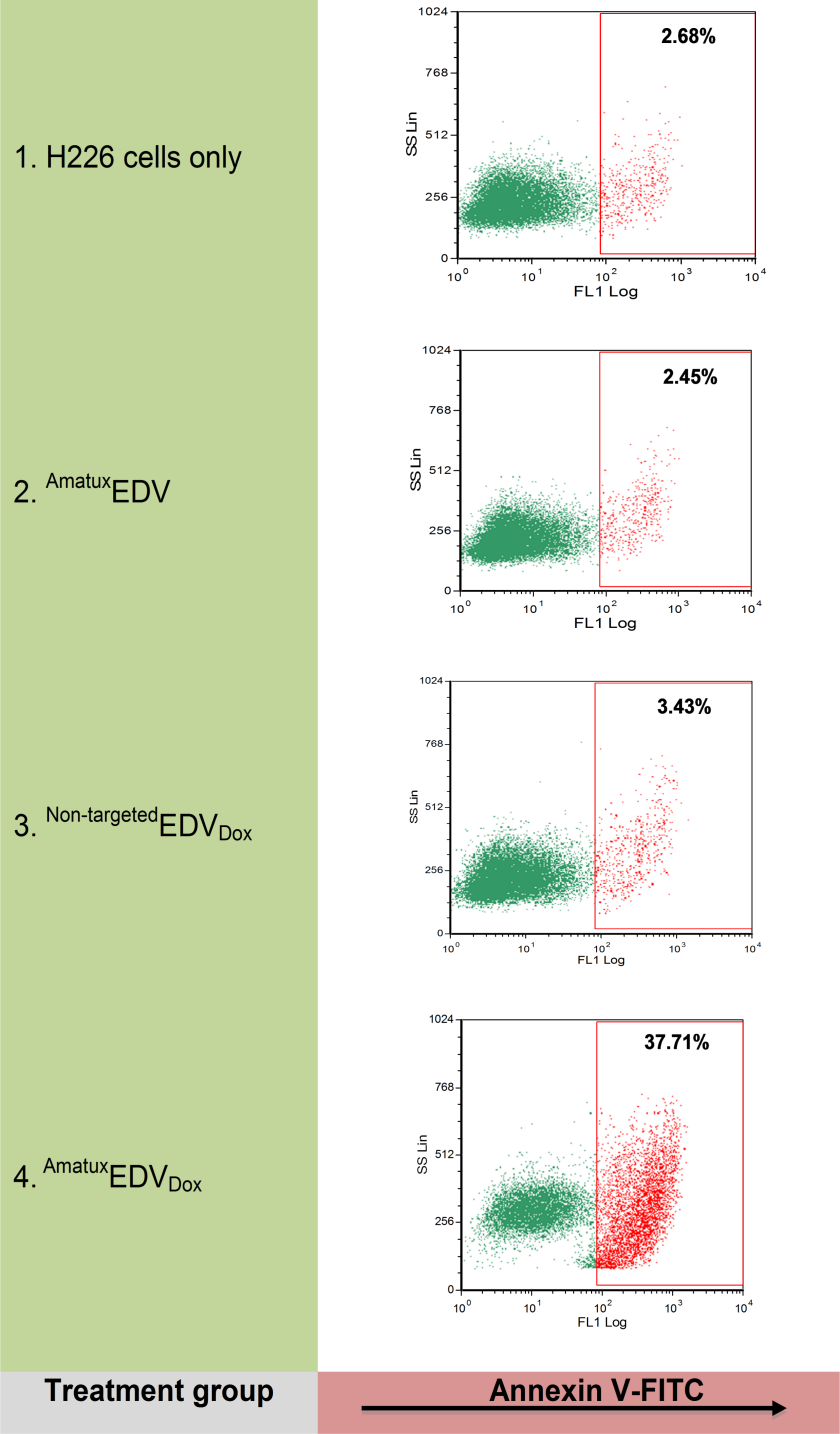
internalisation of ^Amatux^EDV_Dox_ apoptosis induction. The induction of apoptosis due to release of doxorubicin internally was measured using Annexin V-FITC staining, on untreated cells (1), cells treated with ^Amatux^EDV (2), ^Non-targeted^EDV_Dox_ (3), or ^Amatux^EDV_Dox_ (4). Fluorescence was measured using 488 nm laser and 530/30 nm filter. The red dot plot represents Annexin V-FITC positive cells and the green dot plot represents Annexin V-FITC negative cells.

Apoptosis causes externalization of phosphatidylserine from the inner leaflet of the plasma membrane phospholipid bilayer to the cell surface, which can be probed with Annexin V for detection, while living cells remain Annexin V negative [48]. Therefore, we stained ^Amatux^EDV, ^Non-targeted^EDV_Dox_ and ^Amtaux^EDV_Dox_ treated cells with Annexin V-FITC and performed flow cytometry to evaluate the induction of apoptosis. Approximately 37.7% of ^Amatux^EDV_Dox_ targeted H226 cells showed Annexin V positive staining, which was around 9-fold greater than that of ^Amatux^EDV and ^Non-targeted^EDV_Dox_ samples (Fig 4). The same samples were also analysed in the absence of Annexin V; the ^Amatux^EDV_Dox_ sample showed a small increase in fluorescence attributed to the autofluorescence of doxorubicin, which has a comparable emission wavelength to FITC, yet the shift remained within the Annexin V negative gate (data not shown). These results suggest that ^Amatux^EDV_Dox_ nanocells are capable of targeted killing of MSLN-expressing cells. Consequently, tumour regression studies were performed using ^Amatux^EDV_Dox_ nanocells in a H226 cell line xenograft model.

### ^Amatux^EDV_Dox_ nanocells mediate anti-tumour effect in human mesothelioma xenograft models

**Fig 5:**
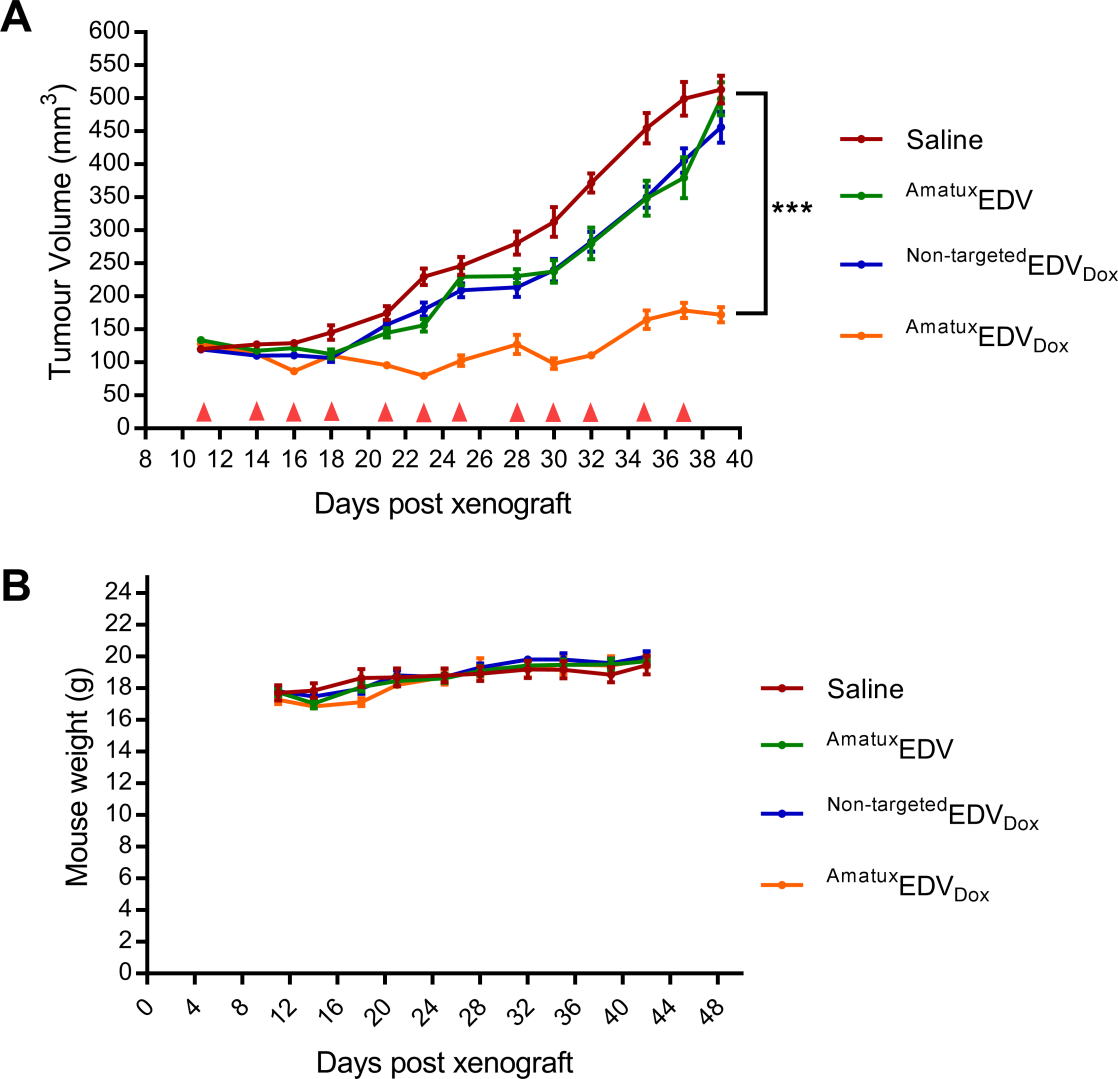
Effect of ^Amatux^EDV_Dox_ on xenograft models. (A) Xenograft implanted mice were treated with either saline as a control, or EDV variants ^Amatux^EDV, ^Non-targeted^EDV_Dox_ or ^Amatux^EDV_Dox_ all at a dose equal to 1 x 10^9^ EDVs at different time points, indicated with a red triangle. The average tumour size at the start is ~110 mm^3^. (B) The mice were weighed twice a week throughout the treatment. Mean ± SEM is shown. (*** = p < 0.001).

Eleven days after tumour subcutaneous-implantation when the volume of the tumours reached ~110 mm^3^, mice were randomly sorted into four different groups (n = 6 per group): saline control group, ^Amatux^EDV, ^Non-targeted^EDV_Dox_ and ^Amatux^EDV_Dox_. By the end of the treatment period (Day 39), all the mice in saline, ^Amatux^EDV and ^Non-targeted^EDV_Dox_ treatment groups had comparable tumour volumes (p > 0.1), while the ^Amatux^EDV_Dox_ treated group showed significant tumour regression (p < 0.001) with the tumour volume being 3-fold less than all other treatment groups (Fig 5A). Within the ^Amatux^EDV_Dox_ treated group, one mouse showed a partial response (≥30% tumour regression) during the whole study period. Three mice showed transient partial response followed by slight tumour growth and then tumour stasis (no tumour growth or progressive disease). One mouse demonstrated stable tumour size during the whole treatment course (data not shown). The ^Non-targeted^EDV_Dox_ did not evoke any significant anti-tumour effect through passive targeting. Thus, active targeting through BsAbs is essential for the efficient delivery of EDV nanocells to tumours.

All mice survived throughout the treatment period, except one mouse in the ^Amatux^EDV_Dox_ group that died of unknown cause(s) on the first day of the experiment, thus n = 5 in subsequent analysis of data for this group. Physical observation of all the mice showed no abnormalities in behaviour nor physical condition, and there was no difference in weight throughout the treatment period between the groups of mice (Fig 5B). This suggests the doxorubicin-loaded EDV nanocells are well tolerated despite the high and repeated dose regimen.

### ^Amatux^EDV_Dox_ nanocells mediate anti-proliferative effect and reduce necrosis in human mesothelioma xenograft models

**Fig 6:**
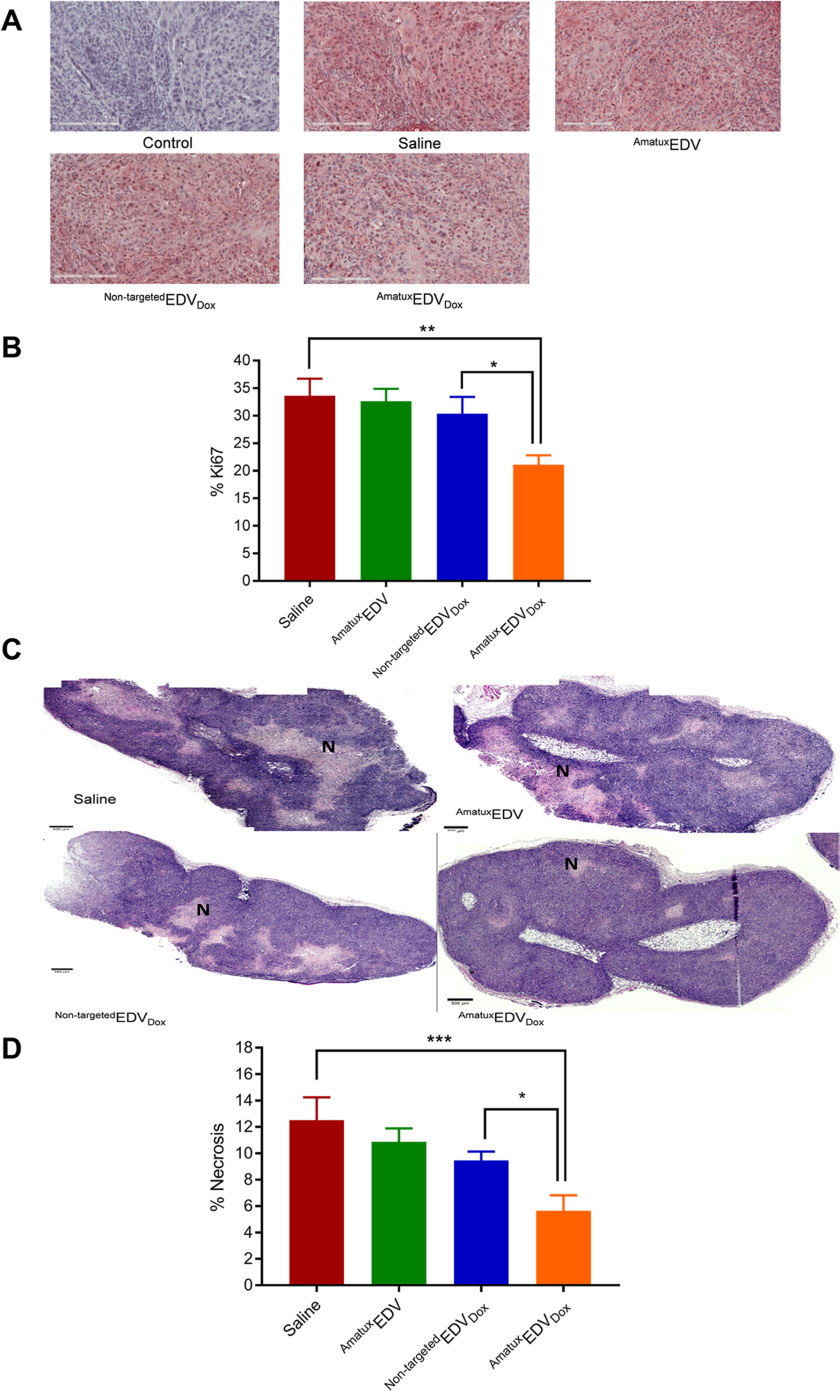
Immunohistochemical staining of paraffin embedded mesothelioma xenograft tumour samples. (A) Malignant mesothelial cells showed strong and diffuse staining by anti-human pKi67 antibody. Tissue stained with secondary antibody only showed no staining. Scale bar 200 μm. (B) The percentage of Ki67-positive cells were calculated blindly from tumour tissue sections obtained from three different mice representing each group (Mean ± SD). (C) Tumour necrotic areas in the tissue sections are shown by reduced H&E staining. Representative tissue sections for the four treatment groups show necrotic areas (N) (absence of nuclear staining). Scale bar 500 μm. (D) The percentage of necrosis was calculated blindly using ImageJ from tumour tissue sections obtained from three different mice representing each group (Mean ± SD). (* = p < 0.05, ** = p < .01 and *** = p < 0.0005).

The nuclear protein Ki67 is a proliferation marker and an independent prognostic factor in malignant mesothelioma that can be utilised to evaluate the effectiveness of a chemotherapy regimen [49, 50]. The Ki67 biomarker was therefore utilised to detect the proliferation rate differences between the four treatment groups. The Ki67 immunohistochemical staining of tissue sections from excised xenograft tumours from the ^Amatux^EDV_Dox_ treated group showed a significant decrease in the number of proliferating cells, compared to the saline (p < 0.01), ^Amatux^EDV (p < 0.01) and ^Non-targeted^EDV_Dox_ (p < 0.05) treated groups (Fig 6A and 6B). Using H&E staining, tissue sections from saline treated mice revealed larger areas of necrosis (12.52%) compared to ^Amatux^EDV (10.9%), ^Non-targeted^EDV_Dox_ (9.5%) and ^Amatux^EDV_Dox_ (5.65%) treatment groups (Fig 6C and 6D). Statistically, the ^Amatux^EDV_Dox_ treated mice had significantly smaller necrotic areas compared to the saline group, ^Amatux^EDV and ^Non-targeted^EDV_Dox_ (p ≤ 0.0005, p < 0.01 and p < 0.05, respectively).

## Discussion

Human malignant mesothelioma is an intractable tumour that develops from the mesothelial cells mainly as a result of asbestos exposure. Challenges exist for current mesothelioma treatment protocols due to the lack of specificity of cytotoxic drugs and their low therapeutic index. To address these challenges, we targeted drug-loaded bacterial EDV nanocells to MSLN receptors, which are overexpressed in mesothelioma, to deliver doxorubicin directly to the mesothelioma cells. This was achieved by engineering the MSLN-specific mAb amatuximab as a bispecific antibody, whereby one arm binds the EDV and the other arm binds to tumour cells. Here, we showed the ability of ^Amatux^EDV_Dox_ to suppress the growth of human malignant mesothelioma in xenograft models. EDV nanocells are derived from *Salmonella typhimurium*, and have proven to be effective, safe and well-tolerated in different species [20, 23–25, 27, 51]. This study builds on a body of published research outcomes including clinical research, associated with a nonliving, bacterially-derived drug nanodelivery system [20, 23, 25, 27]. The efficiency of ^BsAb^EDV_Drug_ nanocells as a system that is able to suppress tumours expressing MSLN, is demonstrated.

We assessed the binding of Amatux-LPS and HN1-LPS BsAbs (anti-MSLN-LPS BsAbs) by performing flow cytometry analyses using two mesothelioma cell lines (H226 and MSTO-211H). Amatux-BsAb was able to bind to H226 cells but not to MSTO-211H while HN1-BsAb was unable to bind either cell line (Fig 2C and 2D). This may be a reflection of the lower affinity of the parent HN1 scFv (K_D_ 100 nM) compared with the amatuximab parent scFv (K_D_ 11 nM), and therefore we excluded HN1 scFv from the remainder of experiments. Both BsAbs showed no binding to MSTO-211H; this is most likely due to the low abundance of MSLN on MSTO-211H cells compared with H226 cells as previously reported [52].

scFvs typically demonstrate lower affinity compared to the original bivalent antibody due to a loss of avidity [21, 53–55], but binding of BsAbs to multiple LPS sites on EDVs, forms an antibody corona, creating a highly avid ^BsAb^EDV nanocarrier system [20]. This is evident by the greater binding of the ^Amatux^EDV_Dox_ to H226 cells compared with the Amatux-BsAb (Fig 3C compared with Fig 2C).

In this study, we showed that ^BsAb^EDV_Drug_ nanocells are able to specifically target tumour cells, internalise, release drugs intracellularly and induce apoptosis *in vitro*. Additionally, we showed that treatment of mice containing mesothelioma cell xenografts with ^BsAb^EDV_Drug_ results in suppression of tumour growth. Previously, the ^BsAb^EDV_Drug_ nanocells have been shown to effect significant tumour growth regression in various *in vivo* models [20, 21, 27, 56, 57]. Various chemotherapeutic agents, siRNA and miRNA can be efficiently packaged within the EDV nanocells and delivered to cancer cells [20, 27, 51]. These nanocells have a greater capacity to package drugs (<10 million drug molecules) [20], when compared with other systems such as liposomes and antibody-drug conjugates, which are only able to be packaged with ~10,000 drug molecules per liposome and <10 drugs molecules per antibody, respectively [58]. Packaging of drug molecules within EDV nanocells allows a significantly lower overall dose compared to systemic drug delivery and as a consequence less potential side effects. For example, the doxorubicin dose was reduced around 1764-fold with ^CD3^EDV_Dox_ compared to that of conventional chemotherapy treatment [20].

Rapidly growing malignant tumours are characterised by poor and fragile vascularisation that results in low oxygen levels which leads to necrosis; thus large, malignant tumours have greater levels of necrosis [59–61]. In mesothelioma, necrosis is a sign of tumour growth [62, 63]. We were able to identify necrotic areas in xenograft tumour sections, and observed that tumours from mice treated with ^Amatux^EDV_Dox_ showed significantly smaller areas of necrosis compared to ^Non-targeted^EDV_Dox_ (p < 0.05). This likely reflects the difference between the two treatment groups in terms of tumour size, with the larger tumours showing more necrosis. Thus, targeted delivery of encapsulated doxorubicin to mesothelioma cells caused tumour growth retardation leading to smaller necrotic areas compared to the other treatment groups. Combined with the results obtained from staining the proliferation marker Ki67, which showed that cells within the tumour sections from ^Amatux^EDV_Dox_-treated mice had significantly lower proliferation, we have demonstrated that ^Amatux^EDVs can be used to target mesothelioma cells and deliver therapeutic doses of doxorubicin, resulting in reduced cell proliferation, lower cellular necrosis and suppression of tumour growth.

## Conclusions

We showed that the full-length antibody amatuximab can be engineered into a BsAb format, which is capable of binding doxorubicin-loaded nanocells, forming ^Amatux^EDV_Dox_. These targeted nanocells are functional and able to bind MSLN receptors on the surface of H226 cells *in vitro* and deliver doxorubicin intracellularly, resulting in the induction of apoptosis. We also showed that the new drug-delivery system ^Amatux^EDV_Dox_ is able to deliver and release doxorubicin in mesothelioma xenografts and suppress the growth of malignant cells. These results indicate that MSLN expression is retained in implanted xenografts and the protein is accessible by the ^BsAb^EDV. MSLN abundance on tumour cells is sufficient for efficient antibody targeting of doxorubicin-loaded EDVs to xenografts, resulting in suppression of tumour growth as well as reducing cell proliferation as shown by Ki67 staining of excised tumours *ex vivo*. This proof-of-concept animal study provides essential data that the anti-MSLN antibody and the EDV drug delivery system are functional *in vivo* and warrants further translational research of this treatment regime. This is the first investigation associated with usage of MSLN as a target molecule for mesothelioma treatments using doxorubicin-loaded EDV nanocells. Further studies investigating dose optimization, biodistribution and survival rates will be required to obtain a better understanding of this drug delivery system and compare its efficacy to existing malignant mesothelioma treatments.

## Abbreviations

AF-488: Alexa-Fluor 488
BsAb: Bispecific Antibody
Dox: Doxorubicin
EDV: EnGeneIC Dream Vector
GPI: Glycosylphosphatidylinositol
G_4_S: Glycine-Serine linker
LPS: lipopolysaccharide
mAb: Monoclonal Antibody
MDR: Multi-Drug Resistance
MSLN: Mesothelin receptor
scFv: Single-chain variable fragment

## References

[1] Antman KH. Natural history and epidemiology of malignant mesothelioma. Chest. 1993;103(4 Suppl):373S–6S. Epub 1993/04/01. . 8462328 10.1378/chest.103.4_supplement.373s

[2] Muers MF, Stephens RJ, Fisher P, Darlison L, Higgs CM, Lowry E, et al Active symptom control with or without chemotherapy in the treatment of patients with malignant pleural mesothelioma (MS01): a multicentre randomised trial. Lancet. 2008;371(9625):1685–94. doi: 10.1016/S0140-6736(08)60727-8 ; PubMed Central PMCID: PMC2431123. 18486741 10.1016/S0140-6736(08)60727-8 PMC2431123

[3] Stumphius J, Meyer PB. Asbestos bodies and mesothelioma. The Annals of occupational hygiene. 1968;11(4):283–93. . 5721712 10.1093/annhyg/11.4.283

[4] Barrett JC. Cellular and molecular mechanisms of asbestos carcinogenicity: implications for biopersistence. Environmental health perspectives. 1994;102 Suppl 5:19–23. Epub 1994/10/01. ; PubMed Central PMCID: PMC1567260. 7882928 10.1289/ehp.94102s519 PMC1567260

[5] Carbone M, Bedrossian CW. The pathogenesis of mesothelioma. Seminars in diagnostic pathology. 2006;23(1):56–60. Epub 2006/10/19. . 17044196 10.1053/j.semdp.2006.08.002

[6] Mossman BT, Shukla A, Heintz NH, Verschraegen CF, Thomas A, Hassan R. New insights into understanding the mechanisms, pathogenesis, and management of malignant mesotheliomas. The American journal of pathology. 2013;182(4):1065–77. doi: 10.1016/j.ajpath.2012.12.028 ; PubMed Central PMCID: PMC3657618. 23395095 10.1016/j.ajpath.2012.12.028 PMC3657618

[7] Heintz NH, Janssen-Heininger YM, Mossman BT. Asbestos, lung cancers, and mesotheliomas: from molecular approaches to targeting tumor survival pathways. Am J Respir Cell Mol Biol. 2010;42(2):133–9. doi: 10.1165/rcmb.2009-0206TR ; PubMed Central PMCID: PMC2822975. 20068227 10.1165/rcmb.2009-0206TR PMC2822975

[8] Ramazzini C. The global health dimensions of asbestos and asbestos-related diseases. J Occup Health. 2016;58(2):220–3. doi: 10.1539/joh.16-2002-ST ; PubMed Central PMCID: PMCPMC5356970. 27040479 10.1539/joh.16-2002-ST PMC5356970

[9] van Zandwijk N, Clarke C, Henderson D, Musk AW, Fong K, Nowak A, et al Guidelines for the diagnosis and treatment of malignant pleural mesothelioma. Journal of thoracic disease. 2013;5(6):E254–307. doi: 10.3978/j.issn.2072-1439.2013.11.28 ; PubMed Central PMCID: PMC3886874. 24416529 10.3978/j.issn.2072-1439.2013.11.28 PMC3886874

[10] Peto J, Decarli A, La Vecchia C, Levi F, Negri E. The European mesothelioma epidemic. British journal of cancer. 1999;79(3–4):666–72. doi: 10.1038/sj.bjc.6690105 ; PubMed Central PMCID: PMC2362439. 10027347 10.1038/sj.bjc.6690105 PMC2362439

[11] Tada Y, Shimada H, Hiroshima K, Tagawa M. A potential therapeutic strategy for malignant mesothelioma with gene medicine. BioMed research international. 2013;2013:572609 doi: 10.1155/2013/572609 ; PubMed Central PMCID: PMC3581274. 23484132 10.1155/2013/572609 PMC3581274

[12] de Graaf-Strukowska L, van der Zee J, van Putten W, Senan S. Factors influencing the outcome of radiotherapy in malignant mesothelioma of the pleura—a single-institution experience with 189 patients. International journal of radiation oncology, biology, physics. 1999;43(3):511–6. Epub 1999/03/17. . 10078630 10.1016/s0360-3016(98)00409-x

[13] Hazarika M, White RM, Johnson JR, Pazdur R. FDA drug approval summaries: pemetrexed (Alimta). The oncologist. 2004;9(5):482–8. doi: 10.1634/theoncologist.9-5-482 . 15477632 10.1634/theoncologist.9-5-482

[14] van Meerbeeck JP, Gaafar R, Manegold C, Van Klaveren RJ, Van Marck EA, Vincent M, et al Randomized phase III study of cisplatin with or without raltitrexed in patients with malignant pleural mesothelioma: an intergroup study of the European Organisation for Research and Treatment of Cancer Lung Cancer Group and the National Cancer Institute of Canada. Journal of clinical oncology: official journal of the American Society of Clinical Oncology. 2005;23(28):6881–9. Epub 2005/09/30. doi: 10.1200/JCO.20005.14.589 . 16192580 10.1200/JCO.20005.14.589

[15] Vogelzang NJ, Rusthoven JJ, Symanowski J, Denham C, Kaukel E, Ruffie P, et al Phase III study of pemetrexed in combination with cisplatin versus cisplatin alone in patients with malignant pleural mesothelioma. Journal of clinical oncology: official journal of the American Society of Clinical Oncology. 2003;21(14):2636–44. doi: 10.1200/JCO.2003.11.136 . 12860938 10.1200/JCO.2003.11.136

[16] Kelly RJ, Sharon E, Hassan R. Chemotherapy and targeted therapies for unresectable malignant mesothelioma. Lung cancer. 2011;73(3):256–63. doi: 10.1016/j.lungcan.2011.04.014 ; PubMed Central PMCID: PMC3148297. 21620512 10.1016/j.lungcan.2011.04.014 PMC3148297

[17] de Boer PA, Crossley RE, Rothfield LI. A division inhibitor and a topological specificity factor coded for by the minicell locus determine proper placement of the division septum in E. coli. Cell. 1989;56(4):641–9. . 2645057 10.1016/0092-8674(89)90586-2

[18] Ma L, King GF, Rothfield L. Positioning of the MinE binding site on the MinD surface suggests a plausible mechanism for activation of the Escherichia coli MinD ATPase during division site selection. Molecular microbiology. 2004;54(1):99–108. doi: 10.1111/j.1365-2958.2004.04265.x . 15458408 10.1111/j.1365-2958.2004.04265.x

[19] Lutkenhaus J. Assembly dynamics of the bacterial MinCDE system and spatial regulation of the Z ring. Annual review of biochemistry. 2007;76:539–62. doi: 10.1146/annurev.biochem.75.103004.142652 . 17328675 10.1146/annurev.biochem.75.103004.142652

[20] MacDiarmid JA, Mugridge NB, Weiss JC, Phillips L, Burn AL, Paulin RP, et al Bacterially derived 400 nm particles for encapsulation and cancer cell targeting of chemotherapeutics. Cancer cell. 2007;11(5):431–45. doi: 10.1016/j.ccr.2007.03.012 . 17482133 10.1016/j.ccr.2007.03.012

[21] Taylor K, Howard CB, Jones ML, Sedliarou I, MacDiarmid J, Brahmbhatt H, et al Nanocell targeting using engineered bispecific antibodies. mAbs. 2015;7(1):53–65. doi: 10.4161/19420862.2014.985952 . 25523746 10.4161/19420862.2014.985952 PMC4622061

[22] MacDiarmid JA, Madrid-Weiss J, Amaro-Mugridge NB, Phillips L, Brahmbhatt H. Bacterially-derived nanocells for tumor-targeted delivery of chemotherapeutics and cell cycle inhibitors. Cell Cycle. 2007;6(17):2099–105. doi: 10.4161/cc.6.17.4648 . 17786046 10.4161/cc.6.17.4648

[23] MacDiarmid JA, Langova V, Bailey D, Pattison ST, Pattison SL, Christensen N, et al Targeted Doxorubicin Delivery to Brain Tumors via Minicells: Proof of Principle Using Dogs with Spontaneously Occurring Tumors as a Model. PloS one. 2016;11(4):e0151832 doi: 10.1371/journal.pone.0151832 ; PubMed Central PMCID: PMCPMC4822833. 27050167 10.1371/journal.pone.0151832 PMC4822833

[24] Whittle JR, Lickliter JD, Gan HK, Scott AM, Simes J, Solomon BJ, et al First in human nanotechnology doxorubicin delivery system to target epidermal growth factor receptors in recurrent glioblastoma. Journal of clinical neuroscience: official journal of the Neurosurgical Society of Australasia. 2015;22(12):1889–94. doi: 10.1016/j.jocn.2015.06.005 . 26279503 10.1016/j.jocn.2015.06.005

[25] Solomon BJ, Desai J, Rosenthal M, McArthur GA, Pattison ST, Pattison SL, et al A First-Time-In-Human Phase I Clinical Trial of Bispecific Antibody-Targeted, Paclitaxel-Packaged Bacterial Minicells. PloS one. 2015;10(12):e0144559 doi: 10.1371/journal.pone.0144559 ; PubMed Central PMCID: PMCPMC4699457. 26659127 10.1371/journal.pone.0144559 PMC4699457

[26] Karagiannis ED, Anderson DG. Minicells overcome tumor drug-resistance. Nat Biotechnol. 2009;27(7):620–1. doi: 10.1038/nbt0709-620 . 19587665 10.1038/nbt0709-620

[27] MacDiarmid JA, Amaro-Mugridge NB, Madrid-Weiss J, Sedliarou I, Wetzel S, Kochar K, et al Sequential treatment of drug-resistant tumors with targeted minicells containing siRNA or a cytotoxic drug. Nat Biotechnol. 2009;27(7):643–51. doi: 10.1038/nbt.1547 . 19561595 10.1038/nbt.1547

[28] Tang Z, Qian M, Ho M. The role of mesothelin in tumor progression and targeted therapy. Anti-cancer agents in medicinal chemistry. 2013;13(2):276–80. ; PubMed Central PMCID: PMC3568227. 22721387 10.2174/1871520611313020014 PMC3568227

[29] Destro A, Ceresoli GL, Falleni M, Zucali PA, Morenghi E, Bianchi P, et al EGFR overexpression in malignant pleural mesothelioma. An immunohistochemical and molecular study with clinico-pathological correlations. Lung cancer. 2006;51(2):207–15. Epub 2005/12/31. doi: 10.1016/j.lungcan.2005.10.016 . 16384623 10.1016/j.lungcan.2005.10.016

[30] Rump A, Morikawa Y, Tanaka M, Minami S, Umesaki N, Takeuchi M, et al Binding of ovarian cancer antigen CA125/MUC16 to mesothelin mediates cell adhesion. The Journal of biological chemistry. 2004;279(10):9190–8. Epub 2003/12/17. doi: 10.1074/jbc.M312372200 . 14676194 10.1074/jbc.M312372200

[31] Li M, Bharadwaj U, Zhang R, Zhang S, Mu H, Fisher WE, et al Mesothelin is a malignant factor and therapeutic vaccine target for pancreatic cancer. Molecular cancer therapeutics. 2008;7(2):286–96. Epub 2008/02/19. doi: 10.1158/1535-7163.MCT-07-0483 ; PubMed Central PMCID: PMC2929838. 18281514 10.1158/1535-7163.MCT-07-0483 PMC2929838

[32] Bharadwaj U, Li M, Chen C, Yao Q. Mesothelin-induced pancreatic cancer cell proliferation involves alteration of cyclin E via activation of signal transducer and activator of transcription protein 3. Molecular cancer research: MCR. 2008;6(11):1755–65. Epub 2008/11/18. doi: 10.1158/1541-7786.MCR-08-0095 ; PubMed Central PMCID: PMC2929833. 19010822 10.1158/1541-7786.MCR-08-0095 PMC2929833

[33] Bharadwaj U, Marin-Muller C, Li M, Chen C, Yao Q. Mesothelin overexpression promotes autocrine IL-6/sIL-6R trans-signaling to stimulate pancreatic cancer cell proliferation. Carcinogenesis. 2011;32(7):1013–24. Epub 2011/04/26. doi: 10.1093/carcin/bgr075 ; PubMed Central PMCID: PMC3128561. 21515913 10.1093/carcin/bgr075 PMC3128561

[34] Bharadwaj U, Marin-Muller C, Li M, Chen C, Yao Q. Mesothelin confers pancreatic cancer cell resistance to TNF-alpha-induced apoptosis through Akt/PI3K/NF-kappaB activation and IL-6/Mcl-1 overexpression. Molecular cancer. 2011;10:106 Epub 2011/09/02. doi: 10.1186/1476-4598-10-106 ; PubMed Central PMCID: PMC3175472. 21880146 10.1186/1476-4598-10-106 PMC3175472

[35] Chang MC, Chen CA, Hsieh CY, Lee CN, Su YN, Hu YH, et al Mesothelin inhibits paclitaxel-induced apoptosis through the PI3K pathway. The Biochemical journal. 2009;424(3):449–58. Epub 2009/09/15. doi: 10.1042/BJ20082196 . 19747165 10.1042/BJ20082196

[36] Cheng WF, Huang CY, Chang MC, Hu YH, Chiang YC, Chen YL, et al High mesothelin correlates with chemoresistance and poor survival in epithelial ovarian carcinoma. British journal of cancer. 2009;100(7):1144–53. Epub 2009/03/19. doi: 10.1038/sj.bjc.6604964 ; PubMed Central PMCID: PMC2669998. 19293794 10.1038/sj.bjc.6604964 PMC2669998

[37] Uehara N, Matsuoka Y, Tsubura A. Mesothelin promotes anchorage-independent growth and prevents anoikis via extracellular signal-regulated kinase signaling pathway in human breast cancer cells. Molecular cancer research: MCR. 2008;6(2):186–93. Epub 2008/02/05. doi: 10.1158/1541-7786.MCR-07-0254 . 18245228 10.1158/1541-7786.MCR-07-0254

[38] Hassan R, Ebel W, Routhier EL, Patel R, Kline JB, Zhang J, et al Preclinical evaluation of MORAb-009, a chimeric antibody targeting tumor-associated mesothelin. Cancer immunity. 2007;7:20 ; PubMed Central PMCID: PMC2935758. 18088084 PMC2935758

[39] Chowdhury PS, Viner JL, Beers R, Pastan I. Isolation of a high-affinity stable single-chain Fv specific for mesothelin from DNA-immunized mice by phage display and construction of a recombinant immunotoxin with anti-tumor activity. Proceedings of the National Academy of Sciences of the United States of America. 1998;95(2):669–74. ; PubMed Central PMCID: PMC18478. 9435250 10.1073/pnas.95.2.669 PMC18478

[40] Ho M, Feng M, Fisher RJ, Rader C, Pastan I. A novel high-affinity human monoclonal antibody to mesothelin. Int J Cancer. 2011;128(9):2020–30. doi: 10.1002/ijc.25557 ; PubMed Central PMCID: PMC2978266. 20635390 10.1002/ijc.25557 PMC2978266

[41] Hassan R, Broaddus VC, Wilson S, Liewehr DJ, Zhang J. Anti-mesothelin immunotoxin SS1P in combination with gemcitabine results in increased activity against mesothelin-expressing tumor xenografts. Clinical cancer research: an official journal of the American Association for Cancer Research. 2007;13(23):7166–71. Epub 2007/12/07. doi: 10.1158/1078-0432.CCR-07-1592 . 18056197 10.1158/1078-0432.CCR-07-1592

[42] Chowdhury PS, Pastan I. Analysis of cloned Fvs from a phage display library indicates that DNA immunization can mimic antibody response generated by cell immunizations. J Immunol Methods. 1999;231(1–2):83–91. . 10648929 10.1016/s0022-1759(99)00142-8

[43] Hassan R, Kindler HL, Jahan T, Bazhenova L, Reck M, Thomas A, et al Phase II clinical trial of amatuximab, a chimeric antimesothelin antibody with pemetrexed and cisplatin in advanced unresectable pleural mesothelioma. Clinical cancer research: an official journal of the American Association for Cancer Research. 2014;20(23):5927–36. doi: 10.1158/1078-0432.CCR-14-0804 ; PubMed Central PMCID: PMCPMC4252585. 25231400 10.1158/1078-0432.CCR-14-0804 PMC4252585

[44] Hasteh F, Lin GY, Weidner N, Michael CW. The use of immunohistochemistry to distinguish reactive mesothelial cells from malignant mesothelioma in cytologic effusions. Cancer cytopathology. 2010;118(2):90–6. doi: 10.1002/cncy.20071 . 20209622 10.1002/cncy.20071

[45] Pillai K, Pourgholami MH, Chua TC, Morris DL. Ki67-BCL2 index in prognosis of malignant peritoneal mesothelioma. American journal of cancer research. 2013;3(4):411–23. ; PubMed Central PMCID: PMC3744020. 23977450 PMC3744020

[46] Kobayashi M, Huang CL, Sonobe M, Kikuchi R, Ishikawa M, Kitamura J, et al Intratumoral Wnt2B expression affects tumor proliferation and survival in malignant pleural mesothelioma patients. Experimental and therapeutic medicine. 2012;3(6):952–8. doi: 10.3892/etm.2012.511 ; PubMed Central PMCID: PMC3438599. 22969998 10.3892/etm.2012.511 PMC3438599

[47] Fedchenko N, Reifenrath J. Different approaches for interpretation and reporting of immunohistochemistry analysis results in the bone tissue—a review. Diagnostic pathology. 2014;9:221 doi: 10.1186/s13000-014-0221-9 ; PubMed Central PMCID: PMC4260254. 25432701 10.1186/s13000-014-0221-9 PMC4260254

[48] Koopman G, Reutelingsperger CP, Kuijten GA, Keehnen RM, Pals ST, van Oers MH. Annexin V for flow cytometric detection of phosphatidylserine expression on B cells undergoing apoptosis. Blood. 1994;84(5):1415–20. . 8068938

[49] Ghanim B, Klikovits T, Hoda MA, Lang G, Szirtes I, Setinek U, et al Ki67 index is an independent prognostic factor in epithelioid but not in non-epithelioid malignant pleural mesothelioma: a multicenter study. British journal of cancer. 2015;112(5):783–92. doi: 10.1038/bjc.2015.9 ; PubMed Central PMCID: PMC4453963. 25633038 10.1038/bjc.2015.9 PMC4453963

[50] Pillai K, Pourgholami MH, Chua TC, Morris DL. Prognostic significance of Ki67 expression in malignant peritoneal mesothelioma. Am J Clin Oncol. 2015;38(4):388–94. doi: 10.1097/COC.0b013e3182a0e867 . 26214083 10.1097/COC.0b013e3182a0e867

[51] Reid G, Pel ME, Kirschner MB, Cheng YY, Mugridge N, Weiss J, et al Restoring expression of miR-16: a novel approach to therapy for malignant pleural mesothelioma. Annals of oncology: official journal of the European Society for Medical Oncology / ESMO. 2013;24(12):3128–35. doi: 10.1093/annonc/mdt412 . 24148817 10.1093/annonc/mdt412

[52] Scales SJ, Gupta N, Pacheco G, Firestein R, French DM, Koeppen H, et al An antimesothelin-monomethyl auristatin e conjugate with potent antitumor activity in ovarian, pancreatic, and mesothelioma models. Molecular cancer therapeutics. 2014;13(11):2630–40. doi: 10.1158/1535-7163.MCT-14-0487-T . 25249555 10.1158/1535-7163.MCT-14-0487-T

[53] Monnier P, Vigouroux R, Tassew N. In Vivo Applications of Single Chain Fv (Variable Domain) (scFv) Fragments. Antibodies. 2013;2(2):193 doi: 10.3390/antib2020193

[54] Enever C, Batuwangala T, Plummer C, Sepp A. Next generation immunotherapeutics—honing the magic bullet. Curr Opin Biotechnol. 2009;20(4):405–11. doi: 10.1016/j.copbio.2009.07.002 . 19709876 10.1016/j.copbio.2009.07.002

[55] Presta LG. Molecular engineering and design of therapeutic antibodies. Curr Opin Immunol. 2008;20(4):460–70. doi: 10.1016/j.coi.2008.06.012 . 18656541 10.1016/j.coi.2008.06.012

[56] Williams M, Kirschner MB, Cheng YY, Hanh J, Weiss J, Mugridge N, et al miR-193a-3p is a potential tumor suppressor in malignant pleural mesothelioma. Oncotarget. 2015;6(27):23480–95. doi: 10.18632/oncotarget.4346 ; PubMed Central PMCID: PMCPMC4695131. 26125439 10.18632/oncotarget.4346 PMC4695131

[57] Glover AR, Zhao JT, Gill AJ, Weiss J, Mugridge N, Kim E, et al MicroRNA-7 as a tumor suppressor and novel therapeutic for adrenocortical carcinoma. Oncotarget. 2015;6(34):36675–88. doi: 10.18632/oncotarget.5383 ; PubMed Central PMCID: PMC4742203. 26452132 10.18632/oncotarget.5383 PMC4742203

[58] Park JW. Liposome-based drug delivery in breast cancer treatment. Breast cancer research: BCR. 2002;4(3):95–9. doi: 10.1186/bcr432 ; PubMed Central PMCID: PMC138729. 12052251 10.1186/bcr432 PMC138729

[59] Harris AL. Hypoxia—a key regulatory factor in tumour growth. Nature reviews Cancer. 2002;2(1):38–47. doi: 10.1038/nrc704 . 11902584 10.1038/nrc704

[60] Bernards R. Cancer: cues for migration. Nature. 2003;425(6955):247–8. doi: 10.1038/425247a . 13679899 10.1038/425247a

[61] Paton AW, Morona R, Paton JC. Bioengineered microbes in disease therapy. Trends in molecular medicine. 2012;18(7):417–25. doi: 10.1016/j.molmed.2012.05.006 . 22721939 10.1016/j.molmed.2012.05.006

[62] Husain AN, Colby T, Ordonez N, Krausz T, Attanoos R, Beasley MB, et al Guidelines for pathologic diagnosis of malignant mesothelioma: 2012 update of the consensus statement from the International Mesothelioma Interest Group. Arch Pathol Lab Med. 2013;137(5):647–67. doi: 10.5858/arpa.2012-0214-OA . 22929121 10.5858/arpa.2012-0214-OA

[63] Klabatsa A, Sheaff MT, Steele JP, Evans MT, Rudd RM, Fennell DA. Expression and prognostic significance of hypoxia-inducible factor 1alpha (HIF-1alpha) in malignant pleural mesothelioma (MPM). Lung Cancer. 2006;51(1):53–9. doi: 10.1016/j.lungcan.2005.07.010 . 16169121 10.1016/j.lungcan.2005.07.010

